# Identification and Registration for High-Yielding Strain through ST and MLT of *Curcuma caesia* Roxb. (Jor Lab KH-2): A High-Value Medicinal Plant

**DOI:** 10.3390/genes13101807

**Published:** 2022-10-06

**Authors:** Mohan Lal, Sunita Munda, Twahira Begum, Tanmita Gupta, Manabi Paw, Sanjoy Kumar Chanda, Himangshu Lekhak

**Affiliations:** ARD Division, CSIR-North East Institute of Science and Technology (NEIST), Jorhat 785006, Assam, India

**Keywords:** *Curcuma caesia*, AMMI, GGE, high essential oil, MLT, regression

## Abstract

(1) Background: *Curcuma caesia* Roxb. is a high valued crop which is extensively used in pharmaceuticals, flavour and fragrances. *C. caesia* is recognised as an endangered species due to its extensive collection from the wild through human intervention. Therefore, to prevent the species from extinction, it is very necessary to conserve and cultivate this plant species for the sustainable availability of the raw material. (2) Methods: In the present plant breeding programme, a multi-year study was performed for the identification of superior genotypes which will help in conservation. To fulfil this objective, a total of 135 accessions of *C. caesia* were collected from different regions of India and were set up for experimental selection trial for three years (2016–2018). After proper evaluation of the genotypes based on six agronomical traits, five high-yielding genotypes were identified which underwent multilocation trial for two years (2019 and 2020). The stability analysis using the Eberhart–Russell method, AMMI and GGE biplot were used to study the consistency of the genotypes in varied environments compared with the check variety. (3) Results: Analysis of variance indicated significant genotype and environment interaction for the yield traits, i.e., dry rhizome recovery, rhizome yield and essential oil yield. The coefficient of variation (CV) was highest for tillers per plant (21.76) and lowest for the plant height (4.93). All the results clearly demonstrated Jor Lab KH-2 as the highest yielding and stable genotype in varied environments compared with the check variety and other selected genotypes. (4) Conclusions: This genotype was then submitted to ICAR-NBPGR, New Delhi, for germplasm registration and received its confirmation vide registration number INGR 21159. This genotype will greatly benefit the breeders and will also help in the conservation of this endangered species. This is the first report on the identification and registration of a high-yielding variety of *C. caesia*.

## 1. Introduction

*Curcuma caesia* Roxb. commonly known as kala haldi, is named after the ginger family and is the largest family of the order Zingiberales [1]. There are around 1300 species in it, grouped into 53 genera [2]. The genus *Curcuma* is classified under the Zingiberaceae family which consists of more than 80 species, some of which have been used in traditional systems of medicine (Ayurveda, Siddha, Unani) for a long time [3], one of which is *C. caesia*. It is widely distributed in Thailand, Malaysia, Nepal, China, Bangladesh and India. It is native to central and northeast India [4]. It is endangered to Asia’s south-eastern region and rarely found in the Himalayan foothills, Sikkim’s North Hill Forest, and the East Godavari Pappi Hills [5,6,7].

The rhizome of the perennial herb *C. caesia* is bluish-black in colour, hence it is called as black turmeric [7]. The plant has 42 chromosomes and is a diploid species [4,8]. It is usually erect and grows between 0.5 and 1.0 m in height with a short stem. The plant bears pale yellow-coloured, long, tubular flowers, with reddish borders, which are smaller than bracts. It is identified by the presence of a large underground-ovoid bluish-black rhizome. Black turmeric produces a cluster of 10–20 leaves, which are characterised by the presence of a deep violet streak running throughout the leaf lamina. The rhizome is usually 2–6 cm in diameter and has a camphoraceous odour [8]. The rhizome is highly important because of its medicinal properties [9]. The ethnobotanical uses include application of *C. caesia* leaf paste to a scorpion or snake bite, and also the dried powder is mixed with *Andrographis paniculata* and applied to insect and snake bites [10]. Dried leaves and rhizomes are used to treat toothache, wounds, fertility, impotency, vomiting, piles, leprosy, cancer, and allergies [11]. The fresh rhizomes are utilised in leprosy, cancer, epilepsy, vomiting, menstrual disorder, anthelminthic aphrodisiac, and gonorrhoeal discharge [12]. The rhizome of *C. caesia* is mashed into a paste to be used in rheumatic arthritis [13]; also, it is applied for rapid healing after cuts and injuries to control bleeding [14]. The *C. caesia* rhizome is also used to treat tonsillitis [15]. *C. caesia* roots are powdered and taken orally with water during gastric disorder [16]. The rhizome is used as a tonic for brain and heart disease and is also effective against asthma, bronchitis, piles, leukoderma [17], allergic eruptions, epileptic seizures, enlargement of the spleen, tuberculous glands of the neck and tumours [18]. The plasters made from the leaves of the crop are used to treat adenitis, lymphangitis, and furunculosis [8].

The rhizome’s essential oil contains a number of biological benefits [6,19]. It possesses many medicinal properties such as resistance to fungal infection [20], anti-asthmatic activity, acts as relaxant for smooth muscle [21], antioxidant activity [19,22], broncho dilating activity [5], locomotors depressant, CNS depressant and anxiolytic activity [23], anti-bacterial, anthelmintic activity [24], and anti-ulcer activity [25]. The strong odour of the rhizome is due to the presence of the essential oil which is rich in eucalyptol, camphor, and starch, etc. [7]. It contains flavour and fragrances due to the presence of bioactive secondary metabolites which also correlates with the medicinal uses and many important useful pharmaceutical products [26]. The essential oil of *C. caesia* rhizomes contains 30 components, out of which curcumene, bornyl acetate, borneol, elemene, 1, 8-cineole, ar-curcumene, (Z)-ocimene, ar-turmerone and camphor represents the major components constituting 97.48% of the essential oil [27]. Other major components are linalool followed by ocimene, 1- ar-curcumene, zingiberol, 1, 8-cineole and borneol [13]. Eucalyptol (28.55%), camphor (21.73%) and epicurzerenone (19.62%) identified as major components in the rhizome essential oil of *C. caesia* [19]. The bioactive components from the rhizomes are responsible for anti-coagulant, hypoglycaemia, wound healing, anti-inflammatory, anti-oxidative properties, and also exhibits the free radical scavenging property [1,28,29].

Due to widespread habitat destruction caused by human activities, including over utilization of black turmeric for traditional medicine, industrialisation, and urbanisation, *C. caesia* is currently regarded as being vulnerable [6,30]. Therefore, the need of the hour is to identify or develop high-yielding essential oil and rhizome varieties to maintain the sustainable cultivation of these high-value medicinal plant species. The phenotyping of germplasm could be used for the development of new varieties, to enhance the quality parameters in terms of yield, to match genotypes to locations, to help choose the best offspring and parents for breeding and to select more suitable genotypes. The measurement of quantitative or qualitative values for biochemical, physiological, morphological and performance-related aspects is referred to as “plant phenotyping”. These play a vital role in determining quality, growth, and stress resistance traits by serving as observable intermediaries between the environment and gene(s) expression [31]. Plant breeding through selection trial (ST) and multilocation trial (MLT) is the most widely accepted programme for the development or identification of superior variety. Multilocation trial is the most essential criteria of the varietal development which determines the ability of the genotype to perform consistently in different environments, confirming its stability. Because of environmental influences or interactions between the genotype and the environment, the selection in some contexts necessitates the presence of effective selection features [32]. The primary attribute that significantly responds to the two sources of variation is the yield [33]. The Eberhart–Russell method and AMMI model are the most widely accepted analysis for confirmation of stability [34,35]. Additionally, nowadays, GGE biplot analysis is also used by the breeder for the evaluation of stability by ranking the environments and genotypes, making it superior to AMMI biplot analysis [36]. A genotype is considered to be superior if it consistently maintains its high-yielding characteristic across different environments [35,37]. Therefore, the identification of this superior variety will help in the conservation of this species for future prospects and will expand the possibilities for the commercial production of camphor, which is the major component in this crop, and offer the pharmaceutical, flavour and fragrances industries new sources of raw materials. The identified strain Jor Lab KH-2 of *C. caesia* was found to possess high rhizome yield with an average of 124 cm plant height, 49 cm leaf length, 26% dry rhizome recovery, 0.80% essential oil in fresh rhizome weight basis and 26.18 tones/ha rhizome yield. The essential oil of this species has very high commercial values and contains antimicrobial, anti-inflammatory activities and much less genotoxicity [7]. This is the first report on the identification and registration of the superior germplasm of *C. caesia* confirmed through stability analysis which showed consistent and satisfactory agronomical performance.

## 2. Materials and Methods

### 2.1. Germplasm Collection

The collection of *C. caesia* germplasm was carried out in six different states of India, namely, Assam, Arunachal Pradesh, Mizoram, Nagaland, Manipur, and Madhya Pradesh, which led to the accretion of 135 accessions. The accessions were identified by breeder Dr Mohan Lal of the division of Agrotechnology and Rural Development, CSIR-NEIST, Jorhat, India. The germplasm was also submitted to the repository of ICAR-NBPGR, New Delhi, and an Indigenous collection (IC) number was obtained. The herbarium for each genotype was prepared and added to the institute’s departmental herbarium.

### 2.2. Setup of Experimental Trial

The collected accessions were planted in the experimental field with GPS coordinates: longitude 94°9′25.4628″ E, latitude 26°44′15.6948″ N and elevation 94 m over the average sea level. The germplasm was planted in randomised complete block design (RCBD) with spacing of 60 × 60 cm between plant to plant and row to row respectively. The plot size of the field was measured 4 m × 4 m length area. The NPK concentration available in the soil were Nitrogen (269 Kg/ha), P (49 Kg/ha), K (91 Kg/ha). The texture of the experimental soil was sandy loam, pH 5.2. The rhizomes were planted in the month of March 2016 and was harvested after every nine months for the three consecutive years for which the data were recorded.

### 2.3. Evaluation of Selection and Multilocation Trial

The three years (2016, 2017 and 2018) selection trial was performed on the agronomical traits, including number of tillers/plants (TPP), plant height (PH), length of leaf (LL), fresh rhizome yield (FRY), dry rhizome recovery (DRR) and essential oil yield (EO). These six agronomical traits were studied, out of which FRY, DRR and EO are the yield parameters preferred in crop improvement programme. Rhizome and essential oil are the most essential part of the crop due to the presence of bioactive components utilised in the aroma, food and pharmaceutical industries. The data were recorded using ten randomly picked plants of each genotype from each replication. Five high essential oil yielding strains were identified after a three-year selection trial which also yielded high rhizome. The multilocation trial of the selected strains along with one check variety were then conducted at five different locations in northeast India, i.e., Jorhat (Assam), Shillong (Meghalaya), Pasighat (Arunachal Pradesh), Imphal (Manipur) and Lakhamijan (Assam), during the years 2019 and 2020. The check variety under consideration was one of the high rhizome yielding local genotype of Pasighat, Arunachal Pradesh, India, mostly used for commercial cultivation. The environments E1, E2, E3, E4, E5 and E6, E7, E8, E9, E10 depict the locations of Jorhat, Lakhamijan, Imphal, Pasighat and Shillong for the first year and second year, respectively. The trial continued for two more years for the agronomical and quality traits that were considered for the selection trial evaluation.

### 2.4. Isolation and GC-MS Analysis of the Essential Oil

The isolation of essential oil was performed according to the protocol suggested by [38] and modified by [19]. The Clevenger apparatus was used, in which 300 g of fresh rhizome was boiled in 3000 mL of distilled water for 6 h. The isolated essential oil was then collected in a glass vial and excess moisture content was removed by treatment with anhydrous sodium sulphate. The essential oil percentage (*v*/*w*) of the isolated essential oils was estimated using the following formula:

$$\mathrm{Essential}\;\mathrm{oil}\;(\mathrm{FWB})=\frac{volume\;of\;essential\;oil\;isolated\;\left(\mathrm{mL}\right)}{weight\;of\;rhizome\;used\;\left(g\right)}\times 100$$

The TRACE ultra-gas chromatograph equipped with mass spectrophotometer (Thermo Fisher Scientific, Waltham, MA, USA) was used for the GC-MS analysis of the rhizome essential oil. The analysis through GC-MS was as per protocol modified by [19].

### 2.5. Statistical Analysis

Both the pooled and individual analysis of variance (ANOVA) was calculated for the multilocation trial data of the five selected genotypes along with the check variety based on two-year evaluation. The stability models such as Eberhart–Russell, AMMI (additive multiplicative mean interaction model) and GGE (genotype + genotype × environment) was measured to identify the better-performing stable genotypes. A total of ten environments (E1, E2, E3, E4, E5, E6, E7, E8, E9, E10) were studied to check the consistency of the genotype performance by metan R package developed by [39].

## 3. Results

### 3.1. Agronomical Data for MLT of the Five Identified Lines along with the Check

A total of 135 accessions were evaluated for three consecutive years which led to the identification of the five most high-yielding lines for rhizome and essential oil yield. The agronomical data were recorded for each year with five replications. The selected lines were compared with one of the local genotype of NE India denoted by the check variety. The evaluation of the multilocation trial in five different regions of northeast India for two years revealed a mean of 130.28 cm for plant height, tillers per plant (5.36), leaf length (46.41 cm), dry rhizome recovery (20.42%), fresh rhizome yield (19.85 tones/ha) and essential oil (0.61%). The coefficient of variation (CV) was highest for tillers per plant (21.76) followed by essential oil percentage (21.05), while the lowest CV was observed in the plant height (4.93). The maximum plant height (cm) was observed in the check variety (133.48) and minimum in KH-2 (125.68). Similarly, the range for tillers per plant varied from KH-120 (4.84) to KH-71 (5.78); leaf length (cm) from check (43.67) to KH-2 (48.58); dry rhizome recovery (%) from KH-58 (18.74) to KH-2 (25.92); fresh rhizome yield (tones/ha) from KH-100 (18.06) to KH-2 (26.23); and essential oil percentage from the check (0.42) to KH-2 (0.80). The highest and lowest environmental performances for the studied traits were depicted in Table 1.

The maximum environmental performances for the traits of plant height, fresh rhizome yield and essential oil yield were revealed in environment E9, while the best performance of dry rhizome recovery was exhibited in environment E4.

### 3.2. ANOVA of the Agronomical Traits for Six High-Yielding Accessions

The analysis of variance was performed for the MLT of six high-yielding accessions based on joint regression. This analysis clearly indicates that there were significant differences in the genotypes in terms of all the studied traits at *p* > 0.05%. Further, the genotype by environment interaction was exhibited by tillers per plant, dry rhizome recovery, rhizome yield and essential oil yield (Table 2).

The analysis also indicated high significant variation in the check variety and KH-100 at *p* > 0.05% for the essential oil yield. Similarly, for rhizome yield, significant difference was revealed in the genotypes KH-120, KH-71 and the check variety. The traits like leaf length and plant height showed no significant environmental influence on the genotypes.

### 3.3. Stability Analysis through Joint Regression Method

The regression analysis was proposed by Eberhart and Russell, therefore, the model was named after them. In order to observe how the genotypes react differently to various environmental situations, stability parameters such as regression coefficient and deviation from regression were applied. The five identified lines and one control were subject to a stability analysis utilising the Eberhart–Russell (ER) model, which explains a genotype to be stable if it acquires high mean value (b_0_), a regression coefficient (bi) equal to one and a regression from deviation (s^2^di) equal to zero. The stability analysis was conducted for three yield traits, i.e., dry rhizome recovery, fresh rhizome yield and essential oil yield, since a significant genotype and environment interaction was observed in these traits. The maximum DRR was expressed by genotype KH-2 followed by the check variety, but presence of significant regression coefficient in the check variety led to its rejection. The genotype KH-2 showed highest mean value (25.92%), bi = 1.01 and s^2^di = −0.65 showing the values almost similar to that of stable line. Again, for FRY, the highest mean value was expressed in KH-2 followed by the check variety. The genotype KH-2 has a mean value of 26.33 tons/ha, bi = 0.76 and s^2^di = −0.14, compared with the check variety demonstrating mean value 18.92 tons/ha, bi = 0.12 and s^2^di = 0.56 (Table 3).

**Figure 1:**
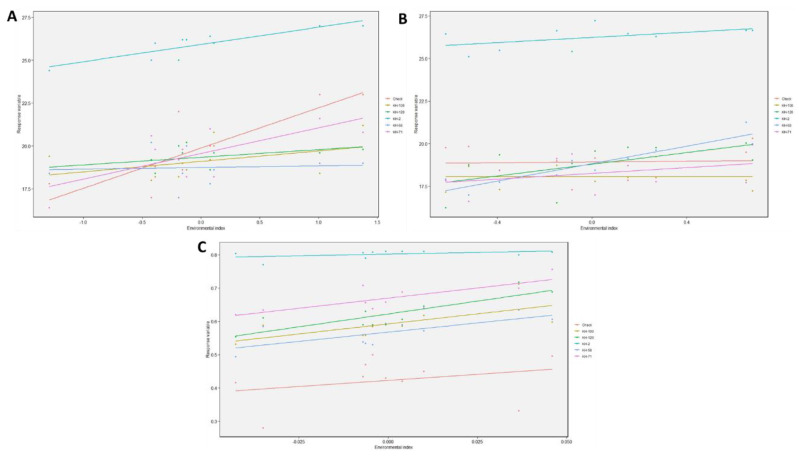
Nominal yield plot for the yield traits (**A**) DRR (**B**) FRY (**C**) EO yield.

The significant deviation from regression in the check variety makes it unsuitable for selection. The highest mean for essential oil yield was observed in KH-2 followed by KH-71 and KH-120 but the regression coefficient of KH-2 was nearer to the standard value of one compared with the other high-yielding genotypes. All these traits confirmed that KH-2 was the most suitable genotype to be considered as the superior line which can perform better in a wide range of environments. The nominal yield plots were also constructed for all the yield traits which clearly indicated that KH-2 was the winning genotype in the entire studied environment, with the highest yield in all the environments (Figure 1).

### 3.4. AMMI ANOVA for the Agronomical Traits for Six High-Yielding Accessions

One of the most widely used AMMI model analyses was also performed to confirm the stability of the identified KH-2 genotype. AMMI ANOVA showed highly significant value for PH, LL, DRR, FRY and EO, indicating wide variation in the studied environment. This makes it necessary to study the consistency of the genotypes because environment can influence the phenotypic nature of the genotype. The genotypes also showed wide variation for all the traits, which was also predicted by the joint regression ANOVA. The environmental influence was observed in all the traits except plant height. The principal component analysis was divided into five principal components: PC1, PC2, PC3, PC4 and PC5. The first principal component correlates strongly with plant height, followed by LL, DRR, FRY, TPP and EO. Similarly, the second principal component corresponds with LL, followed by DRR, FRY, TPP and EO. Furthermore, the third principal component correlates with the traits FRY and EO (Table 4).

### 3.5. Stability Analysis through AMMI Model and GGE Biplot

**Figure 2:**
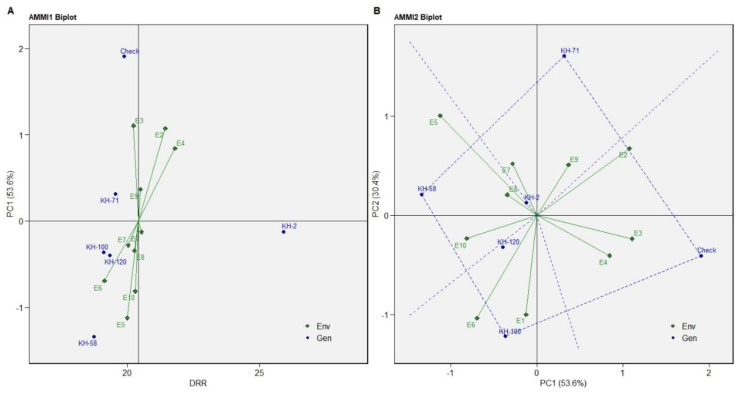
(**A**) AMMI1 biplot (**B**) AMMI2 biplot of DRR of five selected accessions and the check variety of *C. caesia* germplasm for eleven environments.

AMMI biplot 1 and 2 were constructed for the yield traits (DRR, FRY and EO) which indicated KH-2 as the most stable genotype. AMMI1 biplot distinctly indicted KH-2 as the high dry rhizome recovery and stable genotype compared with the other five genotypes (Figure 2A).

**Figure 3:**
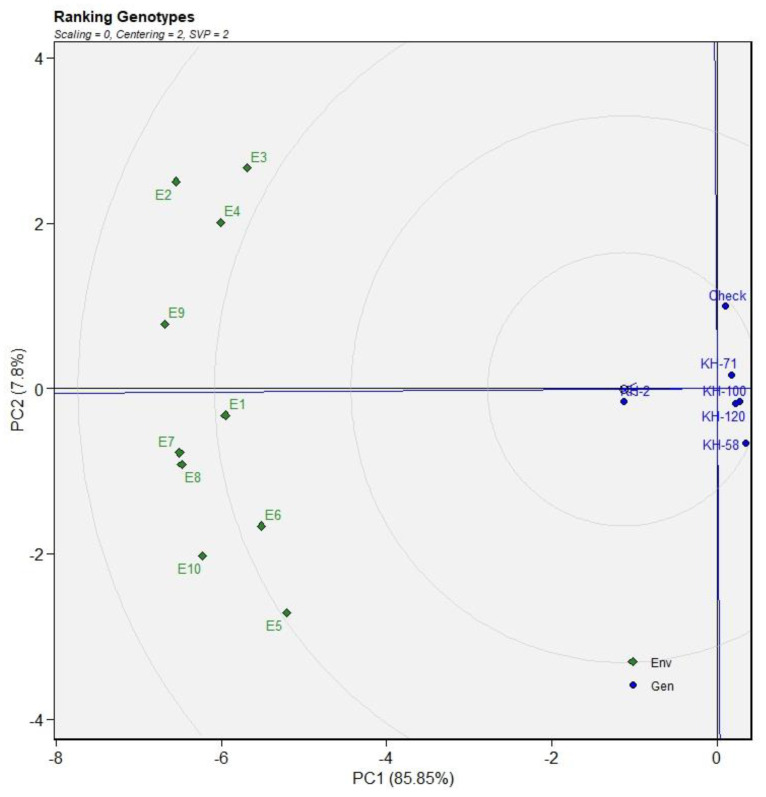
The genotype ranking through GGE plots for dry rhizome yield based on ideal genotype for eleven environments.

AMMI 2 biplot indicated KH-100, KH-58, KH-71 and the check variety as the high-yielding genotypes (Figure 2B). The genotype KH-100 was the most favourable genotype for the environments E1 and E6, while the genotype KH-58 showed better performance in E5, E8 and E10. The best suited genotype for the environments E7 and E9 is KH-71 while the check variety is most favoured in the E2, E3 and E4 environments. Similar results were predicted in GGE plot with KH-2 as the most consistent genotype, followed by KH-71, KH-100, KH-120, KH-58 and the check variety (Figure 3).

**Figure 4:**
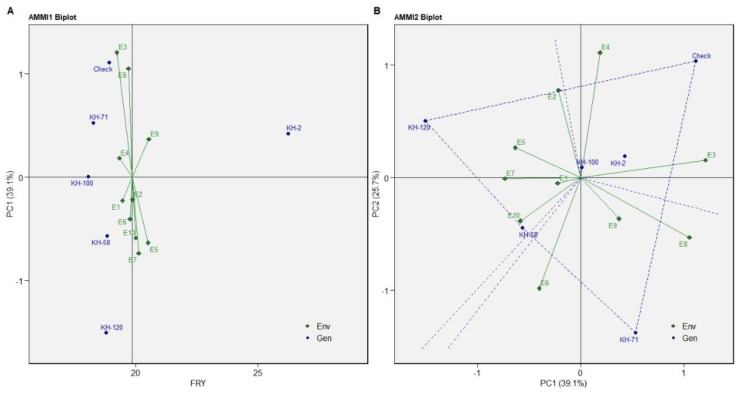
(**A**) AMMI1 biplot (**B**) AMMI2 biplot of FRY of five selected accessions and the check variety of *C. caesia* germplasm for ten environments.

The ideal environment is the centre of the set of concentric lines that functions as a measure to gauge the separation between an environment and the ideal environment. It can be observed that E9 and E7 are the nearest to the ideal environment and therefore can be said to be the most desirable environments. E3 and E5 are the most undesirable studied environments (Figure 3). The trait FRY showed KH-100 as the most stable genotype but with low yield as compared to the other varieties. The genotype KH-2 is the high-yielding genotype for FRY with consistent performance as revealed in the biplot of AMMI1 for FRY (Figure 4A). The genotype KH-71, KH-58, KH-120 and the check variety are the highest yielders for fresh rhizome. The most favourable environments for the check variety are E3 and E4; and environments E6, E8 and E9 for the genotype KH-71. Similarly, the genotypes KH-58 and KH-120 were favourable for the environments E1, E2, E5, E7 and E10 (Figure 4B).

**Figure 5:**
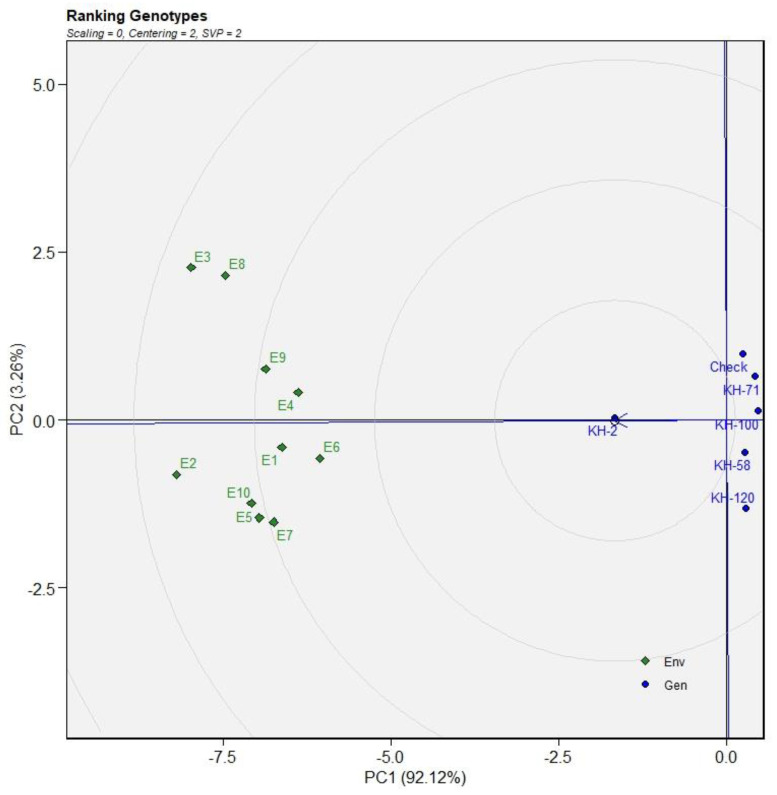
The genotype ranking through GGE plots for FRY based on ideal genotype for eleven environments.

The ideal genotype identified for the trait FRY in the GGE biplot was KH-2. The other genotypes closest to the ideal genotypes were KH-58 and KH-100. The ranking of ideal environments for the trait FRY was E2 followed by E1, E9, E10. The least desirable environments were E3 and E8 (Figure 5).

**Figure 6:**
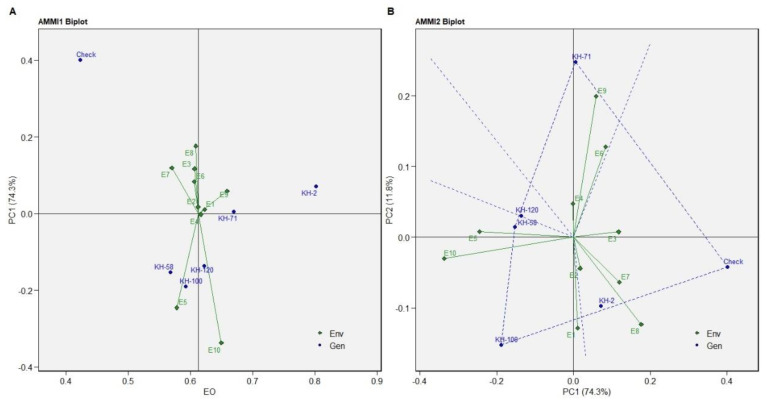
(**A**) AMMI1 biplot (**B**) AMMI2 biplot of EO yield of five selected accessions and the check variety of *C. caesia* germplasm for eleven environments.

Additionally, the AMMI1 biplot for trait EO yield revealed KH-71 and KH-2 as the stable and high essential oil yielding genotypes. However, KH-71 is more stable compared to KH-2 and KH-2 was high-yielding than KH-71. So, both the genotypes can be considered for EO yield (Figure 6A). In the AMMI2 biplot, it can be observed that genotype KH-71, KH-58, KH-100 and the check variety are high essential oil yielding genotypes in different environments (Figure 6B).

**Figure 7:**
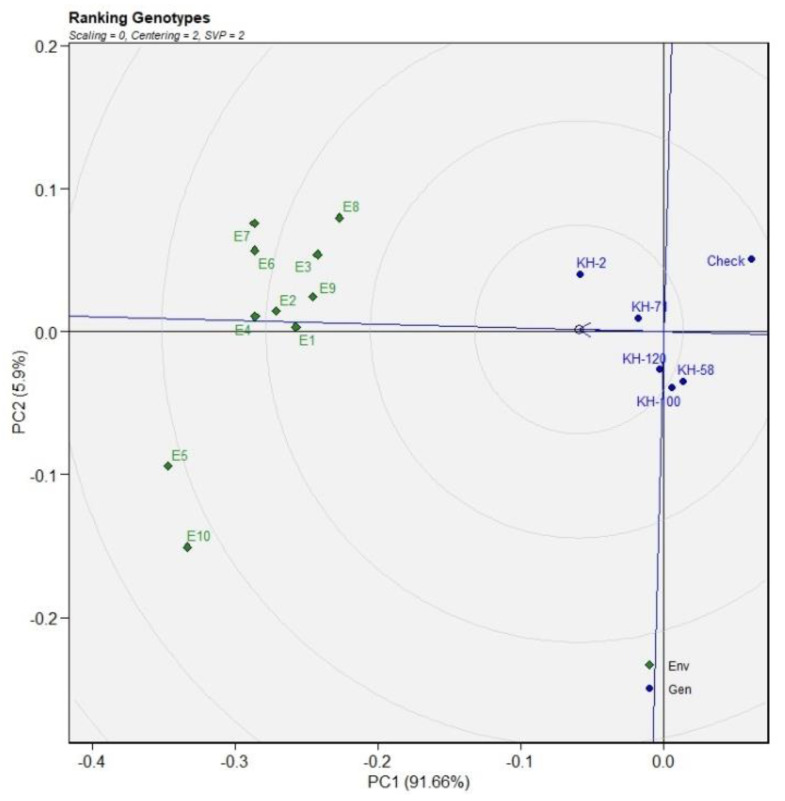
The genotype ranking through GGE plots for EO based on ideal genotype for eleven environments.

The most ideal genotype identified was KH-71, followed by KH-2, KH-120, KH-100 and the check variety, as depicted in the GGE biplot of EO. Environments E4, E2, E1 are the most favourable environments observed according to the GGE biplot of ranking environments for the trait EO (Figure 7).

All the analysis clearly depicts that genotype KH-2 is the most ideal genotype in terms of DRR, FRY and EO yield, maintaining its consistency in a wide range of environments. After the confirmation of the stability and high yield, this identified genotype, named as Jor Lab KH-2, was registered with ICAR-NBPGR, New Delhi, vide registration number INGR 21159.

## 4. Discussion

The expression of superior traits with uniform result is highly desirable. However, the trait expression may differ due to various factors including environment, genotype, and the combined effect of genotype and environment [40,41,42]. This necessitates the need for the study of genotype–environment (G × E) interaction to evaluate the performance and stability for the identification of elite genotypes. Significant genotype–environment interaction was observed for the traits such as tillers per plant, dry rhizome recovery, rhizome yield and essential oil yield, therefore, the study of stability is essential for which a multivariate stability approach, which was used in the study. So far, no previous study has been reported in *C. caesia* for the identification of a stable genotype with superior desirable traits. Therefore, the results obtained in the present study were compared with the other species of Curcuma. The total curcuminoid content in 15 turmeric genotypes for three years was studied representing the major turmeric cultivating regions of India. The stability analysis was performed through the AMMI model, revealing IISR Prathiba and BSR 2 as stable genotypes for bisdemethoxycurcumin content. The genotypes SLP 389/1, Acc. 849 and CO 2 were found to be stable for demethoxycurcumin, while BSR 2, CO 2, IISR Prathiba and Duggirala Red were the genotypes reported to be stable for curcumin content [43]. The findings of the present study reported that genotype by environment interaction was exhibited by tillers per plant, dry rhizome recovery, rhizome yield and essential oil yield for the studied genotypes. A study was conducted on eleven cultivars of turmeric for fresh yield, curcumin yield, curing percentage, and dry yield at five different environments. For fresh yield, a large proportion of variation was attributed to environments (70.8%). For the traits such as curcumin content, curing percentage and dry yield, the variation due to genotype effect accounted for 17.7%, 31.2%, and 15.7%, respectively. The results from G × E interaction using the Eberhart and Russel model reported variation of 42.9% in curcumin content due to environment. For fresh yield with above average yield per plant across all environments, the genotype Mega Turmeric was found as most stable, while, for dry yield across all the environments, the genotypes IISR Prathiba, Mega Turmeric and IISR Kedaram were the ones found to be stable [38,44]. The genotypes by season interactions reported that 25 turmeric genotypes were found to be suitable for selection based on agro-morphological differences. GEIs contributed 34.88% variation in the rhizome yield, variation for weight per plant was 7.04%, 10.97% for rhizome width, the variation for petiole length was 11.95%, 47.25% variation was observed for lamina length and 25.44% variation was observed for lamina width [45]. Evaluation by GGE biplot of seven turmeric genotypes from Nepal was performed to identify traits helpful in turmeric selection. A total of 71.91% variation was observed from the two principal components (PC1 and PC2), among which 39.08% and 32.83% variation was exhibited by PC1 and PC2, respectively [46]. Another study in *Curcuma angustifolia* was carried out to identify a high-yielding stable genotype. The GGE biplot results exhibited the adaptation pattern of genotypes at various environments and the discrimination ability of the environments as well. The results revealed the genotype IGDMT-10-1(G5) as the most stable, and the most suitable environment for all the genotypes was revealed as E5 [47]. In a similar line, the present study revealed the environment E9 and E7 as the most desirable environments. In the environment E9, the traits of fresh rhizome yield, essential oil yield and plant height were found to be best performing, whereas in the environment E4, the dry rhizome recovery was found to be the highest performing trait. Another study was conducted on 17 ginger genotypes from Nigeria to identify stable genotypes and the best environment based on GGE biplot. The results from the GGE biplot model revealed UG2-9-01 as the most stable genotype with better yield for the traits of number of rhizome fingers per plant and rhizome length, while for the best environment, the Ikom environment was revealed as the best discriminating environment [48]. It was also reported that genotype by environment interactions was significant in some yield and growth traits of ginger [49]. The stability analysis for seventeen turmeric genotypes at two locations for two years using the Eberhart and Russel model revealed all the traits studied except oleoresin content and curcumin content to be highly significant for the mean square due to environment and linear. Similarly, all the traits studied except oleoresin content and curcumin content were found to be highly significant for the G × E interactions. Based on the results, stable genotypes with superior yield performance and stable genotypes with high curcumin yield under both favourable and unfavourable conditions were found [50]. A study on the three genotypes of *Curcuma zanthorrhiza* and twenty genotypes of *C. aeruginosa* at three different locations of Indonesia was carried out to evaluate their stability for total phenolic and antioxidant activity. The AMMI and GGE analysis revealed that G1, G6, G13, and G16 were the genotypes identified to be stable for phenolic antioxidant production. Among the identified stable genotypes, three *C. zanthorrhiza* genotypes were found to have higher total phenolic content and antioxidant activity compared to all the genotypes of *C. aeruginosa* in all the studied environments. Moreover, from the biplot analysis, 67.1% and 32.9% variation were found to be exerted by both the principal components (PC1 and PC2), respectively, for total phenolic content, while for the antioxidant activity parameters variation of 89.1% and 10.9% was found to be exerted by PC1 and PC2, respectively [51]. The GGE analysis in the present study confirmed the genotype Jor Lab KH-2 as the most stable genotype with superior yielding traits. A previous report [52,53] revealed that G × E interaction was found to be significant for the trait rhizome yield in turmeric. Another study on seventeen germplasm of turmeric was carried out to study their stability in three environments using the Eberhart and Russell model. The result of ANOVA revealed significant variation for the trait of rhizome yield and all the other thirteen traits studied in all the germplasm in all of the three environments, which accounted for the genetic variation among the studied germplasm. The G × E interaction was found to exhibit significant variation on the traits such as rhizome yield, rhizome thickness, rhizome fresh weight, rhizome dry weight and length of rhizome. CIMCH14130, CIMCH14208 and CIMCH14107 were the three identified stable genotypes with mean values, bi < 1 and s^2^di 0 [54]. Similar results were obtained in our study for the genotype KH-2 with bi = 0.76 and s^2^di = −0.14 while the check variety had bi = 0.12 and s^2^di = 0.56 (Table 4). The result signifies the check variety significantly deviated from regression which highlights that the check variety was not selectable, and the genotype Jor Lab KH-2 was stable with high-yielding traits.

## 5. Conclusions

High crop productivity with consistent performance is the major goal and demand of breeders, researchers and growers. A multi-year study for three years was performed for 135 germplasms of *C. caesia* which led to the selection of five high rhizome yielding lines. These lines were evaluated in a multi-location trial and analysed using various multivariate analyses, such as Eberhart–Russell, AMMI and GGE biplot. The stability test was performed only on yield parameters because the yield parameters are the major goal of the breeder for the improvement of the crop. These analyses helped in the detection of the stable and superior genotype for fresh rhizome yield/plant and essential oil content. This genotype was named as Jor Lab KH-2 and was registered with ICAR-NBPGR, New Delhi. This selected genotype could be utilised for *C. caesia* varietal development programme and could be recommended for commercial cultivation. This superior genotype will also aid in conservation of this rare and endangered species which could be utilised for future prospects. This new variety will expand the possibilities for the commercial production of camphor which is the major component in the rhizome essential oil, and offer the pharmaceutical, flavour and fragrances industries new sources of raw materials which are cost-effective. This is the first report on the identification and registration of a superior variety of *C. caesia* confirmed through stability analysis showing consistent and satisfactory performance.

## Figures and Tables

**Table 1:** Mean and range for all the studied traits of six high-yielding genotypes of *C. caesia* in MLT.

Traits	Mean	SE	SD	CV	MinENV	MaxENV	MinGEN	MaxGEN
PH	130.28	0.37	6.41	4.93	E6 (128.17)	E9 (133.53)	KH-2 (125.68)	Check (133.48)
TPP	5.36	0.07	1.17	21.76	E9 (5.13)	E3 (5.73)	KH-120 (4.84)	KH-71 (5.78)
LL	46.41	0.23	3.9	8.41	E5 (45.52)	E6 (48.78)	Check (43.67)	KH-2 (48.58)
DRR	20.42	0.19	3.32	16.29	E6 (19.13)	E4 (21.8)	KH-58 (18.74)	KH-2 (25.92)
FRY	19.85	0.19	3.36	16.98	E3 (19.23)	E9 (20.52)	KH-100 (18.06)	KH-2 (26.23)
EO	0.61	0.01	0.13	21.05	E7 (0.57)	E9 (0.66)	Check (0.42)	KH-2 (0.8)

PH: plant height; TPP: tillers per plant; LL: leaf length; DRR: dry rhizome recovery; FRY: fresh rhizome yield; EO: essential oil yield, SE: standard error; SD: standard deviation; CV: coefficient of variation; ENV: environment; GEN: genotype.

**Table 2:** ANOVA based on joint regression for the agronomical traits of six high-yielding genotypes of *C. caesia* in MLT.

Source	DF	PH	TPP	LL	DRR	FRY	EO
Total	59	68.87	2.12	35.28	39.30	46.89	0.08
GEN	5	342.63 ***	5.31 ***	157.91 ***	370.12 ***	495.60 ***	0.78 ***
ENV + (GEN × ENV)	54	43.52	1.82	23.93	8.66	5.34	0.01
ENV (linear)	1	776.14	9.16	242.96	150.14	54.60	0.20
GEN × ENV (linear)	5	45.50	4.87 **	37.88	16.68 **	8.74 *	0.01 *
Pooled deviation	48	28.05	1.35	17.91	4.88	3.97	0.01
Check	8	6.80	1.97	24.34 *	8.42	5.42 *	0.02 ***
KH-100	8	36.73	1.26	19.23	5.70	2.95	0.01 ***
KH-120	8	18.86	1.44	20.80	1.83	6.70 *	0.00
KH-2	8	22.61	1.65	9.46	1.04	1.92	0.00
KH-58	8	15.19	1.04	22.52 *	5.02	1.30	0.00 *
KH-71	8	68.11	0.75	11.12	7.27	5.51 *	0.00
Pooled error	200	35.51	1.14	10.72	4.29	2.63	0.00
Total	59	68.87	2.12	35.28	39.30	46.89	0.08

PH: plant height; TPP: tillers per plant; LL: leaf length; DRR: dry rhizome recovery; FRY: fresh rhizome yield; EO: essential oil yield, ENV: environment; GEN: genotype; DF: degree of freedom; *** significant at 0.5%; ** significant at 1%; * significant at 5%.

**Table 3:** Mean and stability parameters for the yield traits of six high-yielding genotypes of *C. caesia*.

GEN	DRR			FRY			EO		
	b_0_	b_i_	s^2^di	b_0_	b_i_	s^2^di	b_0_	b_i_	s^2^di
KH-2	25.92	1.01	−0.65	26.33	0.76	−0.14	0.80	1.11	0.00
KH-58	18.74	0.10 *	0.15	18.83	2.56 ***	−0.27	0.57	0.20 ***	0.00 *
KH-71	19.56	1.49	0.60	18.25	0.85	0.57 *	0.67	1.22	0.00
KH-100	19.10	0.61	0.28	18.06	0.01	0.06	0.59	1.20	0.00 ***
KH-120	19.34	0.45	−0.49	18.79	1.71	0.81 *	0.62	1.54 *	0.00
Check	19.88	2.35 ***	0.83	18.92	0.12	0.56 *	0.42	0.72	0.00 ***

DRR: dry rhizome recovery%; FRY: fresh rhizome yield; EO: essential oil yield; GEN: genotype; *** significant at 0.5%; * significant at 5%; b_0_: mean; b_i_: regression coefficient; s^2^di: regression from deviation.

**Table 4:** AMMI analysis table for different agronomical traits of six high-yielding genotypes of *C. caesia* in the MLT.

Source	DF	PH	TPP	LL	DRR	FRY	EO
ENV	9	86.24 **	1.02	27.00 ***	16.68 ***	6.07 *	0.02 ***
REP(ENV)	40	28.90	1.38	8.28	3.33	2.52	0.00
GEN	5	342.63 ***	5.31 ***	157.91 ***	370.12 ***	495.60 ***	0.78 ***
GEN:ENV	45	34.98	1.98 **	23.31 ***	7.06 **	5.20 ***	0.01 ***
PC1	13	80.09 **	3.34 ***	33.48 ***	13.10 ***	7.05 ***	0.02 ***
PC2	11	21.57	2.47 *	29.51 ***	8.79 *	5.47 *	0.00 *
PC3	9	17.33	1.04	19.86	3.40	5.12 *	0.00 *
PC4	7	16.96	0.98	11.01	1.68	3.42	0.00
PC5	5	4.18	0.50	6.70	1.67	2.46	0.00
Residuals	200	35.51	1.14	10.72	4.29	2.63	0.00
Total	344	40.40	1.44	16.30	10.54	10.55	0.02

PH: plant height; TPP: tillers per plant; LL: leaf length; DRR: dry rhizome recovery; FRY: fry rhizome yield; EO: essential oil yield, ENV: environment; GEN: genotype; DF: degree of freedom; PC: principal component; *** significant at 0.5%; ** significant at 1%; * significant at 5%.
